# Differential responses of gut microbiota to the same prebiotic formula in oligotrophic and eutrophic batch fermentation systems

**DOI:** 10.1038/srep13469

**Published:** 2015-08-25

**Authors:** Wenmin Long, Zhengsheng Xue, Qianpeng Zhang, Zhou Feng, Laura Bridgewater, Linghua Wang, Liping Zhao, Xiaoyan Pang

**Affiliations:** 1 State Key Laboratory of Microbial Metabolism, School of Life Sciences and Biotechnology, Shanghai Jiao Tong University, Shanghai 200240, China; 2 Department of Microbiology and Molecular Biology, Brigham Young University, Provo, Utah 84602, USA; 3 Shanghai Jiao Tong University and Perfect (China) Co. Ltd. Joint Research Center on Microbiota and Health.

## Abstract

The same prebiotics have produced inconsistent effects on microbiota when evaluated in different batch fermentation studies. To understand the reasons behind these discrepancies, we compared impact of one prebiotic formula on the same inoculated fecal microbiota in two frequently used batch systems: phosphate-buffered saline (PBS, oligotrophic) and basal culture medium (BCM, eutrophic). The microbiota was monitored using 454 pyrosequencing. Negative controls (no prebiotic) of both systems showed significant shifts in the microbiota during fermentation, although their pH remained relatively stable, especially in BCM, with increases in *Bilophila* and *Escherichia/Shigella* but a decrease in *Faecalibacterium*. We identified prebiotic responders via redundancy analysis by including both baseline and negative controls. The key positive and negative responders in the two systems were very different, with only 8 consistently modulated OTUs (7 of the 28 positive responders and 1 of the 35 negative responders). Moreover, some OTUs within the same genus responded to the prebiotic in opposite ways. Therefore, to obtain a complete *in vitro* evaluation of the modulatory effects of a prebiotic on microbiota, it is necessary to use both oligotrophic and eutrophic systems, compare treatment groups with both baseline and negative controls, and analyze the microbiota changes down to the OTU level.

The human intestine is colonized by hundreds of beneficial microbial species that are deeply involved in host nutrition, metabolism and immunity^1^. These microbes promote the absorption of certain nutrients and the production of vitamins^2^,^3^, and include bacteria such as *Faecalibacterium* and *Bifidobacterium*, which have been shown to protect hosts from inflammation and infection^4^,^5^. However, a growing body of evidence suggests that opportunistic pathogens in the gut may play a causative role in chronic diseases. For example, an *Enterobacter* strain isolated from the gut of an obese human caused obesity in germ-free mice^6^, and a *Bilophila* strain caused inflammatory bowel disease in IL10^−/−^ germ-free mice^7^. A structurally disrupted gut microbiota with decreased beneficial bacteria and increased detrimental bacteria has been linked to the onset and development of various chronic diseases^8^,^9^.

Targeted modulation of the gut microbiota has thus become a potentially effective strategy to improve host health^10^,^11^. Prebiotics, defined as “non-digestible food ingredients that beneficially affect the host by selectively stimulating the growth and/or activity of one or a limited number of bacterial species already resident in the colon,”^12^ are promising and widely used approaches for modulating gut microbiota. Prebiotics must be non-digestible to pass through the upper GI tract and reach the colon; once there, they stimulate the proliferation or metabolic activity of beneficial bacteria by serving as a substrate for fermentation^13^. Prebiotics have been shown to modulate the composition of the gut microbiota and confer health benefits in both human and animal trials^14^,^15^. For example, consumption of oligofructose in elderly nursing home patients showed a stimulation of *Bifidobacterium* in feces and a diminution of inflammation^16^. Oral administration of inulin-type fructans significantly increased *Bifidobacterium,* prevented high fat diet–induced obesity and improved glucose metabolism in mice^17^.

Potential prebiotics are typically tested using *in vitro* batch fermentation models inoculated with human fecal matter to mimic the human digestive tract environment^15^,^18^. Such studies allow *in vitro* modeling of how the composition of the human gut microbiota changes in response to prebiotic nutrients. However, different batch culture studies have reported inconsistent modulatory effects on the microbiota by the same prebiotics^19^,^20^. Oligofructose, for example, has been widely studied^15^, but only its bifidogenic effects have been reported to be reproducible. The effects on other bacteria, such as *Escherichia*, *Streptococcus*, and *Bacteroides*, have been reported differently from paper to paper—increased in some studies but unchanged or even reduced in others^21^,^22^,^23^.

One possible explanation for these discrepancies is the different culture media used for the *in vitro* systems. The trophic status of the *in vitro* batch fermentation systems that have been utilized varies, but most can be categorized as either “oligotrophic” or “eutrophic.” Oligotrophic systems are inoculated with a higher concentration (typically ranging from 5% to 20%) of fecal slurry as the source of both nutrition and microbiota, with few or no additional nutrients such as vitamins and trace elements^19^,^22^,^24^,^25^. Eutrophic systems, by contrast, are inoculated with a lower concentration of fecal slurry (typically 1%) into a basal medium fortified with peptone, yeast extract and bile salts^20^,^21^,^23^,^26^,^27^,^28^. The phosphate-buffered saline (PBS) system^19^,^22^,^25^ and the basal culture medium (BCM) system^20^,^21^,^26^,^27^ are the two most widely used oligotrophic and eutrophic systems, respectively. Interestingly, these two systems mirror the differences in intestinal trophic status induced by a calorie-restricted diet, in which available nutrients are absorbed primarily while passing through the upper parts of the GI tract (oligotrophic), and a high-fat/high-protein diet, in which excess nutrients reach the colon (eutrophic)^29^,^30^.

A second possible explanation for the discrepancies is the use of different reference controls. Conventionally, a baseline control comprising microbial samples taken at the initial zero-hour time point was used to identify the response of the microbiota to the prebiotic substrates^21^,^22^,^31^. However, a few studies have used negative controls consisting of parallel batch cultures performed in the absence of prebiotics and sampled at various time points alongside the experimental system^19^,^23^.

Recent advances in DNA sequencing technologies have introduced a third possible source of the discrepancy. High-throughput non-targeted next-generation sequencing (NGS) has yielded great advances in microbial ecology, but NGS can also be a source of discrepancy due to different methods of data analysis. Taxon-based analysis at the genus or family level has been widely used for NGS-based profiling of gut microbiota^20^,^32^,^33^,^34^. However, this type of analysis is problematic because accumulating evidence indicates that different species in the same genus may respond in different ways to the same perturbation^35^,^36^,^37^. Thus, merging all the species in the same genus together may overlook real patterns or generate spurious patterns of prebiotic-induced microbial change.

The inconsistent results obtained using *in vitro* tests of potential prebiotics have hampered the study of prebiotics and their impact on microbiota and human health. We therefore undertook this study to assess the impact that different trophic status, different reference controls and microbial DNA sequence data analysis methods have on the outcome of an *in vitro* batch culture study of a prebiotic formula. We aimed to develop an empirical approach to systematically characterize the responses of the microbiota to the evaluated substrates in the *in vitro* batch fermentation models.

## Results

We used 454 pyrosequencing to profile changes in the microbiota during fermentation, with or without the prebiotic formula in the PBS and BCM systems, in samples collected at 0, 6, 24 and 72 h. A total of 50,073 usable pyrosequencing reads were obtained from the 16 samples. After discarding sequences that had no near neighbors in the entire Greengenes database, we used a total of 50,056 reads (average of 3130 sequences per sample) for downstream analysis (Supplementary Fig. S1a). Operational taxonomic units (OTUs) were delineated at the 98% similarity level because higher thresholds generated a dramatic increase in the OTU numbers, which might represent microdiversity at the subspecies level (Supplementary Fig. S1b). A total of 550 OTUs (average of 201 OTUs per sample) were generated from all the samples (Supplementary Fig. S1c). Rarefaction analysis and the Shannon Diversity Index (H’) based on the abundance of the representative OTU sequences revealed that, although new rare phylotypes would be expected with additional sequencing, most of the diversity had already been captured (Supplementary Fig. S1d,e).

**Figure 1:**
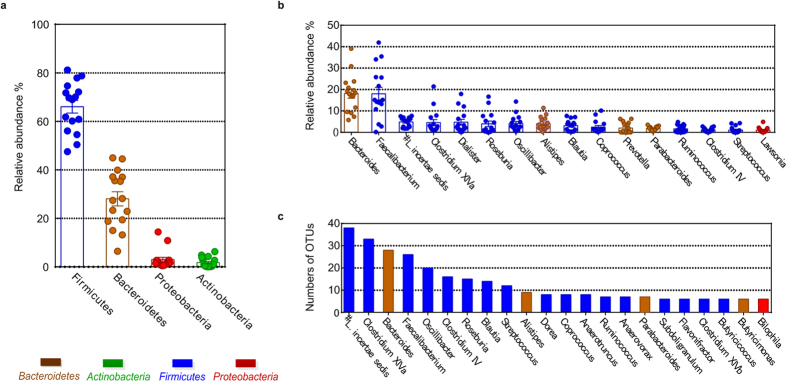
The taxa-level gut microbiota composition of the fermentation samples. (**a**) Relative abundance of the four predominant phyla in the fermentation samples. (**b**) The sixteen dominant genera (each >1% of the total sequences), shown with the relative abundance of these genera in each sample. (**c**) The twenty-two genera that contained more than 5 OTUs each.

Across all the samples, 98.87% and 87.00% of the total sequences were assigned to different phyla and genera, respectively. *Firmicutes*, *Bacteroidetes, Proteobacteria* and *Actinobacteria* were the 4 dominant phyla in all the samples (contributing 66.56% (427 OTUs), 27.44% (65 OTUs), 3.10% (26 OTUs) and 1.77% (22 OTUs) of the total sequences, respectively) (Fig. 1a). At the genus level, 401 OTUs were classified into 80 genera. Sixteen of the 80 genera each occupied more than 1% of the total sequences, including *Bacteroides* (17.66%), *Faecalibacterium* (17.46%), *Lachnospiraceae incertae sedis* (5.02%), *Clostridium* XIVa (4.73%), and *Dialister* (4.52%) (Fig. 1b). Twenty-two of the 80 genera had more than 5 OTUs, including *Lachnospiraceae incertae sedis* (38 OTUs), *Clostridium* XIVa (33 OTUs), *Bacteroides* (28 OTUs)*, Faecalibacterium* (26 OTUs), and *Oscillibacter* (20 OTUs) (Fig. 1c).

### Variations of microbiota in negative controls

**Figure 2:**
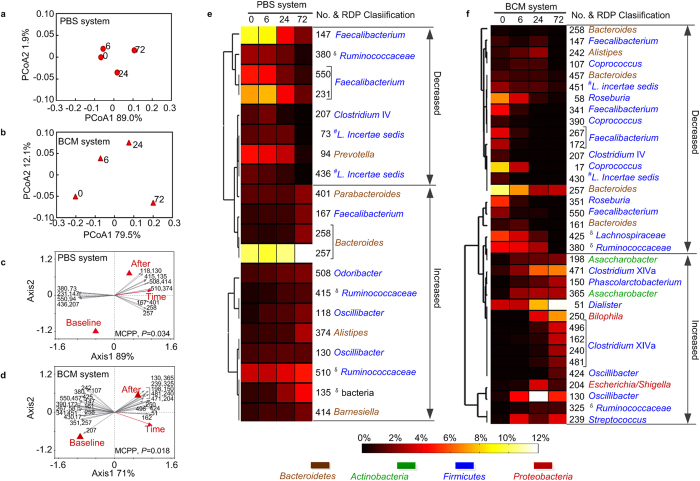
Variations of microbiota in negative controls of the PBS and BCM systems. (**a,b**) Principal coordinate analysis (PCoA) score plots based on the weighted UniFrac distances, in PBS (**a**) and BCM (**b**), respectively. (**c,d**) Biplot of the key identified OTUs according to redundancy analysis on log 10-transformed relative abundance of OTUs in PBS (**c**) and BCM (**d**). Constrained explanatory variables are indicated by red triangles and red arrow. The P-value of the Monte Carlo Permutation Procedure (MCPP) is shown at lower right. (**e,f**) Heat map of the relative abundance of the 20 OTU-level phylotypes in PBS (**e**) and the 35 OTU-level phylotypes in BCM (**f**), identi**f**ied as key variables for differentiation between the microbiota structure of baseline and after fermentation at 6, 24 and 72 hours. The OTUs are arranged according to their co-occurrence clusters based on Spearman correlation coefficients. The deepest level of confident taxonomic annotation of these OTU lineages was obtained by the Ribosomal Database Project classifier, ^**δ**^means unclassified, ^*#*^*L.* means *Lachnospiraceae* (see legend at the bottom for color key). The increased or decreased differentiation of the key phylotypes after fermentation is determined according to redundancy analysis.

In the negative controls of both the PBS and BCM systems, pH remained largely unchanged (Supplementary Fig. S2). However, principal coordinate analysis (PCoA) based on the relative abundance of OTUs revealed that the microbiota shifted over time in both negative control cultures. In the PBS system, marked changes in the microbiota were first observed at 24 h and continued thereafter (Fig. 2a), whereas in the BCM system, the inoculated microbiota changed rapidly within the first 6 h of fermentation and showed continued change at 24 h and 72 h (Fig. 2b). Redundancy analysis was employed to characterize the increase or decrease of phylotypes in the negative control PBS and BCM systems during fermentation. The baseline (comprising 0 h of both negative control and prebiotic cultures) and after-fermentation (6, 24, 72 h negative control cultures) groups were set as nominal constrained explanatory variables, and the incubation time was set as non-nominal. The log 10-transformed relative abundances of OTUs (with more than 1% in at least one sample) were used as response variables.

In the negative control of the PBS system, the Monte Carlo Permutation procedure (MCPP) showed that the constrained ordination model was significant (*P* = 0.034), and 89% of the variance could be explained by the first canonical axis. We identified 20 key shifted OTUs that had at least 50% of the variability in their values explained by the first axis (Fig. 2c). Of these 20, 8 OTUs decreased after fermentation, mainly *Faecalibacterium* (3 OTUs), *Clostridium* IV (1 OTU), *Lachnospiraceae incertae sedis* (2 OTUs) and *Prevotella* (1 OTU). The other 12 OTUs increased, mainly *Faecalibacterium* (1 OTU), *Oscillibacter* (2 OTUs), *Odoribacter* (1 OTU), *Bacteroides* (2 OTUs), *Parabacteroides* (1 OTU), *Alistipes* (1 OTU) and *Barnesiella* (1 OTU) (Fig. 2e, Supplementary Table S1).

In the negative control of the BCM system, MCPP also showed that the constrained ordination model was significant (*P* = 0.018). Thirty-five OTUs with at least 50% of the variability in their values explained by the first axis were identified as the key shifted OTUs during fermentation over time (Fig. 2d). Of these 35, 20 OTUs decreased, mainly *Faecalibacterium* (5 OTUs)*, Coprococcus* (3 OTUs), *Roseburia* (2 OTUs), *Clostridium* IV (1 OTU), *Bacteroides* (4 OTUs) and *Alistipes* (1 OTU). The other 15 OTUs increased, including *Clostridium* XIVa (5 OTUs), *Oscillibacter* (2 OTUs), *Phascolarctobacterium* (1 OTU), *Streptococcus* (1 OTU), *Dialister* (1 OTU), *Bilophila* (1 OTU), *Escherichia/Shigella* (1 OTU) and *Asaccharobacter* (2 OTUs) (Fig. 2f, Supplementary Table S2).

### Variations of microbiota during prebiotic fermentation in the PBS system

**Figure 3:**
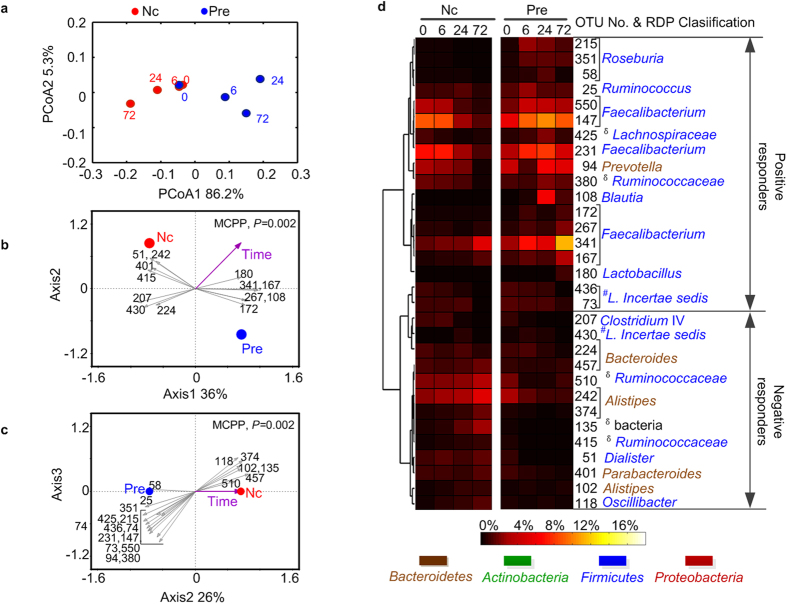
Variations of microbiota during prebiotic fermentation in the PBS system. “Pre” means Prebiotic cultures, and “Nc” means negative control cultures. (**a**) Princip**a**l coordinate analysis (PCoA) score plots based on the weighted UniFrac distances. (**b, c**) Biplot of the key identified OTUs according to redundancy analysis on log 10-transformed relative abundance of OTUs. Constrained explanatory variables are indicated by red and blue circles and the purple arrow. P-value of the Monte Carlo Permutation Procedure (MCPP) is shown in the upper right. (**b**) Plot of first and second axes. (**c**) Plot of second and third axes. (**d**) Heat map showing relative abundance of the 31 OTU-level phylotypes, identified as key variables for differentiation between the microbiota structure of “Pre” and “Nc” over time. OTUs are arranged according to their co-occurrence clusters based on Spearman correlation coefficients. The deepest level of confident taxonomic annotation of these OTU lineages was obtained by the Ribosomal Database Project classifier, ^δ^means unclassified, ^*#*^*L.* means *Lachnospiraceae* (see legend at the bottom for color key). The increased or decreased differentiation of the key phylotypes after fermentation with the prebiotic is determined according to redundancy analysis and is marked “positive responders” or “negative responders,” respectively.

We observed that the pH values decreased during the first 6 h of microbial fermentation with the addition of the prebiotic, from an initial pH of 6.5 to 3.8 (6 h), 3.7 (24 h) and 3.8 (72 h) (Supplementary Fig. S3a). A PCoA score plot based on weighted-UniFrac distance showed that fermentation with the prebiotic changed the overall microbiota in a direction opposite that of the negative controls, mainly on the PC1 axis (which explained 86.2% of the variation) (Fig. 3a).

To identify the key phylotypes positively or negatively responding to the prebiotic, redundancy analysis was performed through constrained modeling with incubation time as consecutive variables and prebiotic vs. negative control as nominal. MCPP showed that the microbiota of prebiotic cultures was significantly segregated from that of the negative controls (*P* = 0.002), and 52% of the variance in OTU abundance data can be explained by the first and second canonical axes. Thirty-one OTUs were identified as positive or negative responders to the prebiotic (Fig. 3b,c). The 18 enriched OTUs belonged mainly to the genera of *Faecalibacterium* (7 OTUs)*, Roseburia* (3 OTUs), *Blautia* (1 OTU), *Lactobacillus* (1 OTU) and *Prevotella* (1 OTU). The 13 inhibited OTUs distributed mainly across *Dialister* (1 OTU), *Oscillibacter* (1 OTU), *Clostridium* IV (1 OTU), *Bacteroides* (2 OTUs), *Parabacteroides* (1 OTU) and *Alistipes* (3 OTU). (Fig. 3d, Supplementary Table S3).

### Variations of microbiota during prebiotic fermentation in the BCM system

**Figure 4:**
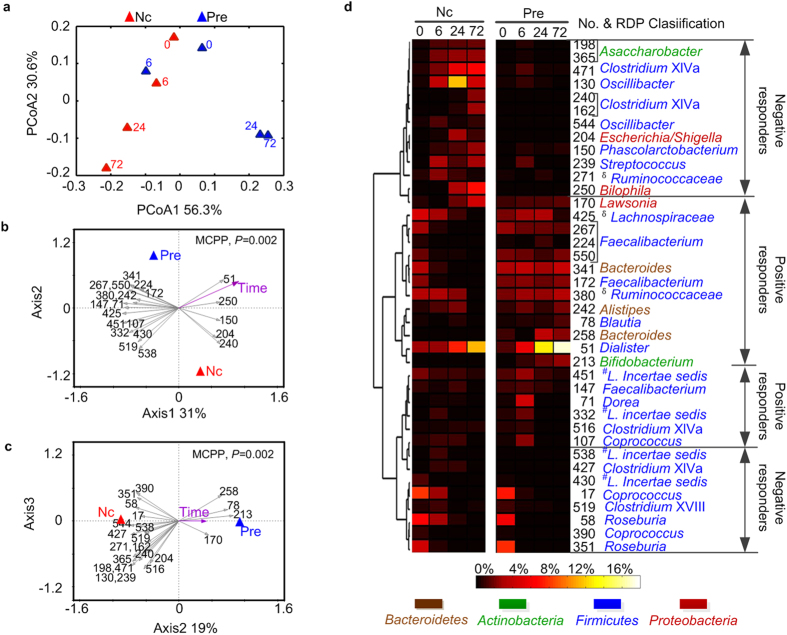
Variations of microbiota during prebiotic fermentation in the BCM system. “Pre” means Prebiotic cultures, and “Nc” means negative control cultures. (**a**) Princip**a**l coordinate analysis (PCoA) score plots based on the weighted UniFrac distances. (**b,c**) Biplot of the key identified OTUs according to redundancy analysis on log 10-transformed relative abundance of OTUs. Constrained explanatory variables are indicated by red and blue circles and red arrow. P-value of the Monte Carlo Permutation Procedure (MCPP) is shown in the upper right. (**b**) Plot of first and second axes. (**c**) Plot of second and third axes. (**d**) Heat map showing relative abundance of the 39 OTU-level phylotypes, identified as key variables for differentiation between the microbiota structure of “Pre” and “Nc” over time. The OTUs are arranged according to their co-occurrence clusters based on Spearman correlation coefficients. The deepest level of confident taxonomic annotation of these OTU lineages was obtained by the Ribosomal Database Project classifier, ^**δ**^means unclassified,^*#*^*L.* means *Lachnospiraceae* (see legend at the bottom for color key). The increased or decreased differentiation of the key phylotypes after fermentation with the prebiotic is determined according to redundancy analysis and is marked as “positive responders” or “negative responders,” respectively.

The pH values decreased in the first 6 h of microbial fermentation in BCM with the addition of the prebiotic, from an initial pH of 7.1 to 4.3 (6  h), 3.8 (24 h) and 3.7 (72 h) (Supplementary Fig. S3b). A PCoA score plot based on weighted-UniFrac distance showed that the overall microbiota was changed after fermentation with the prebiotic in a direction opposite that of the negative controls, primarily on the PC1 axis (which explained 56.3% of the variation) (Fig. 4a).

Redundancy analysis demonstrated that the overall microbiota from samples of the prebiotic were significantly different from that of the negative control cultures over time (*P* = 0.002, MCPP), and 50% of the variance can be explained by the first two canonical axes. Thirty-nine OTUs were identified as responding OTUs to the prebiotic (Fig. 4b,c). Of these 39, 17 increased OTUs belonged mainly to *Faecalibacterium* (5 OTUs), *Coprococcus* (2 OTUs), *Lachnospiraceae incertae sedis* (2 OTUs), *Dialister* (1 OTU), *Blautia* (1 OTU), *Bacteroides* (2 OTUs) and *Bifidobacterium* (1 OTU). The other 22 decreased OTUs were distributed mainly across *Clostridium* XlVa (5 OTUs), *Roseburia* (2 OTUs), *Oscillibacter* (2 OTUs), *Coprococcus* (2 OTUs), *Streptococcus* (1 OTU), *Escherichia/Shigella* (1 OTU), *Bilophila* (1 OTU) and *Asaccharobacter* (2 OTUs) (Fig. 4d, Supplementary Table S4).

### Comparison of microbial responses to the prebiotic in the two systems

**Figure 5:**
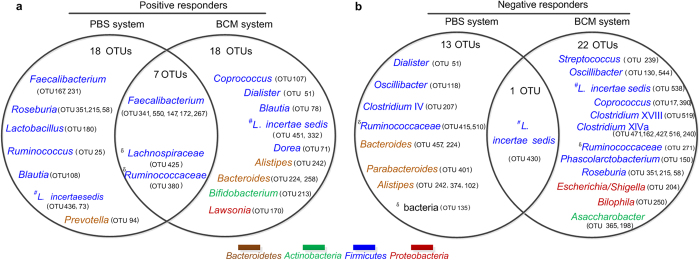
Venn diagrams of key responding phylotypes to the prebiotic in the PBS and BCM systems. (**a**) Positive responders. (**b**) Negative responders. The deepest level of confident taxonomic annotation of these OTU lineages was obtained by the Ribosomal Database Project classifier, ^**δ**^means unclassified,^*#*^*L.* means *Lachnospiraceae* (see legend at the bottom for color key).

The key phylotypes that responded to the same prebiotic in PBS and BCM systems were very different. A total of 28 OTUs were identified as positive responders in PBS and/or BCM system. Of these 28, only 7 OTUs—OTU341, 550, 147, 172 and 267 in *Faecalibacterium*, OTU425 in unclassified *Lachnospiraceae* and OTU380 in unclassified *Ruminococcaceae*—were consistently stimulated in both systems. OTUs in *Bifidobacterium*, *Lactobacillus*, *Roseburia*, *Prevotella*, *Bacteroides* or *Dialister* were stimulated by the prebiotic in just one system. However, 35 OTUs were identified as negative responders in the PBS and/or BCM systems. Of these, only 1 OTU (OTU430, *Lachnospiraceae incertae sedis*) was consistently inhibited in both systems. Thirty-four OTUs were inhibited by the prebiotic in only one system, including *Bacteroides*, *Alistipes* and *Oscillibacter* in PBS and *Bilophila*, *Escherichia/Shigella*, and *Streptococcus* in BCM. Three OTUs—OTU51 (*Dialister*), OTU224 (*Bacteroides*) and OTU242 (*Alistipes*)—responded oppositely to the prebiotic formula in the two systems: increasing in BCM but decreasing in PBS (Fig. 5a,b).

Notably, several OTUs that belong to the same genus responded oppositely to the prebiotic in the same culture system. Several OTUs in *Coprococcus* (OTU107, 17 and 390) provide an example; OTU107 was stimulated by the prebiotic in the BCM system, whereas OTU17 and OTU390 were inhibited. Key OTUs distributed across the genus of *Lachnospiraceae incertae sedis* also showed opposite responses to the prebiotic formula (Fig. 5a,b). Additionally, opposite changes among species within the same genus occurred even in the negative control cultures. For example, during fermentation in the negative control PBS culture, three predominant *Faecalibacterium* phylotypes (OTU147, 550, 231) decreased, whereas a low-abundance phylotype of *Faecalibacterium* (OTU167) increased (Fig. 2e).

## Discussion

*In vitro* batch modeling of the digestive tract is particularly useful for evaluating the effects of potential prebiotic substrates on microbiota^15^,^18^. However, the same prebiotics have been reported to exert different modulatory effects on gut microbiota in different studies. This study was undertaken to investigate possible sources of the discrepancies. Our results indicate that different trophic status of the culture medium, the use of baseline vs. negative controls as the point of reference, and species-specific responses of gut bacteria to prebiotics are key contributors to these discrepancies.

The trophic status of the culture medium may be the most important factor influencing the growth of bacteria in the *in vitro* fermentation model. Rycroft *et al.* reported that the prebiotic oligofructose significantly stimulated *Bifidobacterium*, *Bacteroides*, *Streptococcus*, *Clostridia* and *Escherichia coli* when evaluated in the BCM^21^. By contrast, Wang *et al.* showed that oligofructose exerted a preferential stimulatory effect on *Bifidobacterium* but kept populations of *Escherichia coli* and *Clostridium* at low levels in the PBS system^22^. In the present study, we showed marked differences in the microbial modulatory effects exerted by the same prebiotic in PBS and BCM cultures, and even the negative control cultures conducted in the absence of prebiotic showed striking differences in microbiota structure under the two different culture conditions. Thus, the different nutritional availability in oligotrophic and eutrophic fermentation systems has a major impact on the growth of the inoculated microbiota in either the presence or absence of a prebiotic substrate.

The higher availability of peptides and bile salts in the BCM culture system may be the major reason for different modulatory effects of the prebiotic, because each of these ingredients alone has been shown to significantly modulate the microbiota composition. Walker *et al.* reported that increasing the concentration of peptides in a culture system significantly stimulated *Bacteroides* and inhibited *Bifidobacterium* and *Clostridium* cluster XIVa + b^38^. MacFarlane *et al.* reported that the proteolytic species in the large bowel were distributed primarily across the genera *Bacteroides*, *Propionibacterium*, *Clostridium*, *Fusobacterium*, *Streptococcus* and *Lactobacillus*^39^. Gut bacteria are also differentially tolerant of bile salts. For instance, Lopez-Siles *et al.* reported 8 *Faecalibacterium prausnitzii* isolates that were all bile-sensitive, with most of the strains showing decreased growth in the presence of the lowest tested concentration (0.1%) of bile salts^40^, whereas *Bacteroides* spp. and *Enterococcus* spp. could tolerate up to 10% and 40% bile salt concentrations, respectively^41^. In our study, *Faecalibacterium* was depleted in BCM negative control systems with 0.05% bile salts. When the prebiotic was added to the BCM system, Faecalibacterium maintained its initial population level, dropping a hint that the prebiotic may antagonize the inhibitory effect of the bile salts on *Faecalibacterium*, which need to be verified in prospective studies. In the PBS system, which contained limited bile salts, addition of the prebiotic significantly increased *Faecalibacterium*. Therefore, pre-existing nutrients such as peptides and bile salts may have a significant impact on how the microbiota is modulated by a prebiotic substrate.

Peptides and bile salts can be detected in the feces of all healthy humans, but their available concentration in the colon differs between individuals owing to differences in diet^42^,^43^,^44^,^45^. A Western diet that is high in animal fats and proteins results in relatively more bile salts and protein reaching the large intestine^44^. Cumming *et al.* showed that a 2.5-fold increase in the amount of animal fat in the diet significantly increased excretion of total fecal bile acids (2.3-fold, on average) in healthy humans^29^. The authors also demonstrated that increasing dietary protein intake by 2.0-fold resulted in a significantly increased ammonia concentration (2.1-fold, on average) in human feces^30^. Ammonia is produced from the fermentation of proteins by bacteria^46^. Our BCM culture system was fortified with at least a 2-fold higher concentration of peptides and bile salts than that in the PBS culture system and may therefore reflect the intestinal trophic status on a high-fat/high-protein diet. Conversely, our PBS culture system was more similar to the intestinal trophic status produced by a normal or calorie-restricted diet, in which a greater fraction of the nutrients consumed are utilized before reaching the colon.

The dysbiotic features of microbiota in our BCM culture system were similar to those observed in human and animal trials of high-fat/high-protein diets. In a study comparing rural African children who consumed a diet relatively high in plant-based nutrients with European children who consumed a relatively high-fat/high-protein diet, the fecal microbiota of the latter showed significantly more *Enterobacteriaceae* (*Shigella*/*Escherichia*) and less *Faecalibacterium*^47^. David *et al.* reported that healthy persons placed on an animal-based diet rapidly and reproducibly produced a fecal microbial community with an increase of bile-tolerant microorganisms (*Bilophila*, *Escherichia, Alistipes* and *Bacteroides*) and a decrease of short-chain fatty acid (SCFA)-producing bacteria (*Lachnospiraceae*, *Eubacterium* and *Coprococcus*)^44^. Cani *et al.* reported that administration of a high-fat diet stimulated *Enterobacteriaceae* but inhibited *Bifidobacterium* in mice^48^. Devkota *et al.* showed that consumption of a diet high in saturated fat promoted the expansion of a low-abundance, sulfite-reducing pathobiont, *Bilophila wadsworthia*, in both wild-type and IL10^−/−^ mice^43^. In our BCM negative control cultures, *Escherichia/Shigella* and *Bilophila* were stimulated and butyrate-producing bacteria, including *Faecalibacterium* and *Roseburia,* were depleted over time. Conversely, in our PBS negative control cultures, *Escherichia/Shigella* and *Bilophila* remained at a low abundance, comparable to that observed in the feces of humans on a normal or calorie-restricted diet. Thus, the two different trophic systems used in batch fermentation cultures may mimic the trophic conditions of the gut on different diets. It is therefore necessary to use both of these systems when evaluating the modulatory effects of a potential prebiotic substrate on gut microbiota *in vitro*.

The use of baseline vs. negative controls is the second potential source of discrepancy in the published literature. Baseline controls have conventionally been performed to evaluate the modulatory effects of prebiotic substrates on the microbiota, while negative controls have often been neglected, presumably because of their stable pH and minimal production of total SCFAs after batch incubation of fecal microbiota without prebiotic carbohydrates^49^, which might indicate a lack of fermentation^21^. In the present study, however, we observed that, despite stable pH curves within negative control cultures, marked microbial changes occurred in both systems. For example, in the eutrophic BCM negative control, several predominant *Faecalibacterium* species decreased and *Bilophila, Escherichia/Shigella* and *Streptococcus* increased. Therefore, the use of only baseline controls to evaluate the effects of a prebiotic may produce perplexing or misleading results. For example, it had been widely demonstrated that inulin can stimulate the amount of *Faecalibacterium prausnitzii* in human trials^50^,^51^; this was also shown in a *Faecalibacterium prausnitzii* mono-culture study^52^. However, it was also reported that inulin significantly decreased the amount of *Faecalibacterium prausnitzii* in BCM *in vitro* batch culture evaluations^53^,^54^,^55^. This incongruity may be due to the use of BCM systems with comparisons to baseline controls only for the *in vitro* evaluations.

In this study, in the BCM system, the prebiotic formula showed no effect on several predominant *Faecalibacterium* species when compared with the baseline, but it stimulated *Faecalibacterium* species when compared with negative controls. Use of a baseline control accounts for the way bacterial communities change over time in culture, and use of a negative control allows determination of which changes are due to the prebiotic. Therefore, inclusion of negative controls is essential for evaluating the modulatory effects of prebiotics *in vitro*.

The last potential source of discrepancies we examined is the data-mining techniques utilized for next-generation sequencing data. At present, most published studies use higher taxon-based analysis, in which OTUs are grouped together and data are examined at genus or even higher taxonomic levels to generate the pattern of response to the prebiotic^21^,^56^. In this study, we found that not all OTUs in the same genus responded to the prebiotic. Furthermore, different OTUs in the same genus (such as *Coprococcus* and *Lachnospiraceae incertae sedis*) displayed different responses to the same prebiotic perturbation. Opposite responses of two OTUs in the same genus could mask each other, making them difficult or impossible to detect by taxon-level analysis, possibly explaining why the effects of prebiotics on a genus are often controversial in the literature. It is thus necessary to use OTU-based analytical methods that reveal species-level structural changes of the microbiota to avoid spurious results and to maximize detection of prebiotic-induced changes.

In conclusion, our study shows that the trophic status of the batch fermentation model, the choice of reference control, and the DNA sequence analysis methodology all play pivotal roles in revealing the effects of a prebiotic substrate on microbiota *in vitro*. To obtain a complete picture of the modulatory effects of a prebiotic on microbiota and to ensure that different laboratories can compare their results, we recommend a standard protocol that utilizes both oligotrophic and eutrophic culture conditions, both baseline and negative controls, and OTU-based analysis of the microbiota sequencing data.

## Methods

### Preparation of PBS and BCM batch culture systems

The PBS medium contained NaCl at 8 g l^−1^, KCl at 0.2 g l^−1^, Na_2_HPO_4_ at 1.15 g l^−1^, KH_2_PO_4_ at 0.2 g l^−1^, L-Cysteine at 0.05%, adjusted to pH 7.3). The BCM medium contained peptone water at 2 g l^−1^, yeast extract at 2 g l^−1^, NaCl at 0.1 g l^−1^, K_2_HPO_4_ at 40 mg l^−1^, KH_2_PO_4_ at 40 mg l^−1^, MgSO_4_·7H_2_O at 10 mg l^−1^, CaCl_2_﹒6H_2_O at 10 mg l^−1^, NaHCO_3_ at 2 g l^−1^, L-cysteine at 0.05%, bile salts at 0.5 g l^−1^, vitamin K at 10 μl l^−1^, Tween 80 at 2 ml l^−1^ and hemin at 5 mg l^−1^, adjusted to pH 7.4^57^.

A fresh fecal sample was collected from a healthy woman, age 27, who had no known metabolic or gastrointestinal diseases and had taken no antibiotics or prebiotics for three months prior to the study. Written informed consent was obtained from this donor. The 10% (w/v) fecal slurry was prepared by diluting the fecal sample in sterile PBS medium, thoroughly suspended^57^, and placed into an anaerobic chamber (H_2_:CO_2_:N_2_, 10:10:80, Whitley DG500 anaerobic work station, Don Whitley Scientific, West Yorkshire, UK) within 30 min after collection. After being filtered through two layers of gauze, the fecal solution was inoculated into the BCM and PBS batch culture systems.

The total volume of each culture system was 20 ml. The PBS system was started with 5% fecal inoculum added with 10 ml of 10% fecal slurry, and the BCM system was started with 1% fecal inoculum added with 2 ml of 10% fecal slurry. Negative control cultures consisted of culture medium and inoculum but no prebiotic substrate. Prebiotic cultures consisted of culture medium, inoculum and a prebiotic formula (2.5%, w/v), which was a mixture of galactooligosaccharide and guar gum at a ratio of 1:1 (w/w). Cultures of negative control and prebiotic formula were performed in the PBS and BCM systems, respectively, in an anaerobic chamber at 37 °C without stirring, and samples were dynamically collected at 0, 6, 24 and 72 h in both systems and both prebiotic and negative control cultures. This study was approved by the School of Shanghai Jiao Tong University Ethics Committee Biomedical Project (document no. 2014-018), and all experiments were performed in accordance with the relevant guidelines and regulations.

### pH measurement

Dynamic culture samples were refrigerated at –80°C until all were collected. Culture liquids (2 ml) were centrifuged at 9000 *g* for 5 min to extract culture supernatants for pH measurement (Mettler Toledo, Columbus, OH, USA).

### Bar-Coded 454 pyrosequencing of the 16S rRNA gene V1-V3 region

The following primers were used to amplify the V1-V3 region of each sample for pyrosequencing: forward primer PF002, 5′-CGTATCGCCTCCCTCGCGCCATCAG-ACGCTCGACA-AGAGTTTGATYMTGGCTCAG-3′; and reverse primer PRxxx, 5′-CTATGCGCCTTGCCAGCCCGCTCAG-NNNNNNNNNN-ATTACCGCGGCTGCTGG-3′. The bar-coded 10-base ID tag in the reverse primer was used to distinguish PCR products from different samples.

For each sample, a 25 μl PCR mix was prepared containing 10 × Pfx amplification buffer (Invitrogen, USA), 0.3 mM dNTP mix, 0.5 mM MgSO_4_ (Invitrogen, USA), 0.25 μM forward primer PF002, 0.25 μM reverse primer PRxxx, 0.25 U Platinum® Pfx DNA polymerase (Invitrogen, USA) and 10 ng template DNA. Temperature cycling included an initial 3 min denaturation at 95 °C; 21 cycles of 1 min at 95 °C, 30 sec at an annealing temperature that dropped 0.5 °C every cycle from 65 °C to 55 °C, and 1 min at 72 °C; 4 more cycles with annealing at 55 °C and 6 min at 72 °C. PCR products were mixed in equal amounts for pyrosequencing as described previously^35^.

### Bioinformatics analysis of pyrosequencing

All raw reads were sorted into different samples according to the sample-unique 10-base barcodes. High-quality sequences were selected using the following criteria: 1) 3′ ends were trimmed when the average Phred quality score of a sliding window of 50 nt dropped below 25, 2) forward primer was detected 3) sequence length ranged from 300 to 700 nt, and 4) sequences contained no more than 2 N bases in the variable region. High-quality sequences were extracted and aligned in Greengenes to remove sequences with less than 75% identity with bacteria. OTU classification and taxonomic assignments were performed using QIIME (v1.2.1)^58^. The most abundant sequence of each OTU was selected as the representative sequence and analyzed by RDP Classifier for taxonomical assignment with a bootstrap cutoff of 50%. Richness and diversity were estimated using rarefaction analysis and the Shannon Diversity Index (H’) based on abundance of the representative OTU sequences (Supplementary Fig. S1d, S1e). Redundancy analysis models were constructed to identify OTUs that distinguished pairs of treatment groups, with Canoco for Windows 4.5 (Microcomputer Power, Ithaca, NY, USA) according to the manufacturer’s instructions. A co-occurrence clustering method based on Spearman correlation coefficients of the selected responder OTUs identified clusters of OTUs that responded similarly to prebiotic treatment.

### Sequence data accession numbers

The pyrosequencing reads described in this study have been deposited in the sequence read archive (SRA) at the NCBI under the accession numbers SRP059185.

## Additional Information

**How to cite this article**: Long, W. *et al.* Differential responses of gut microbiota to the same prebiotic formula in oligotrophic and eutrophic batch fermentation systems. *Sci. Rep.* **5**, 13469; doi: 10.1038/srep13469 (2015).

## Supplementary Material

Supplementary Information
