# Supplementary material for: Differential responses of gut microbiota to the same prebiotic formula in oligotrophic and eutrophic batch fermentation systems
Source: Sci Rep. 2015 Aug 25;5:13469. doi: 10.1038/srep13469 (PMC4548253; doi:10.1038/srep13469)
Supplement: Supplementary Information [file srep13469-s1.pdf]

Supplementary Information

Differential responses of gut microbiota to the same prebiotic formula in oligotrophic and eutrophic batch fermentation systems

Wenmin Long, Zhengsheng Xue, Qianpeng Zhang, Zhou Feng, Laura Bridgewater, Linghua Wang, Liping Zhao, Xiaoyan Pang

Supplementary figures and tables

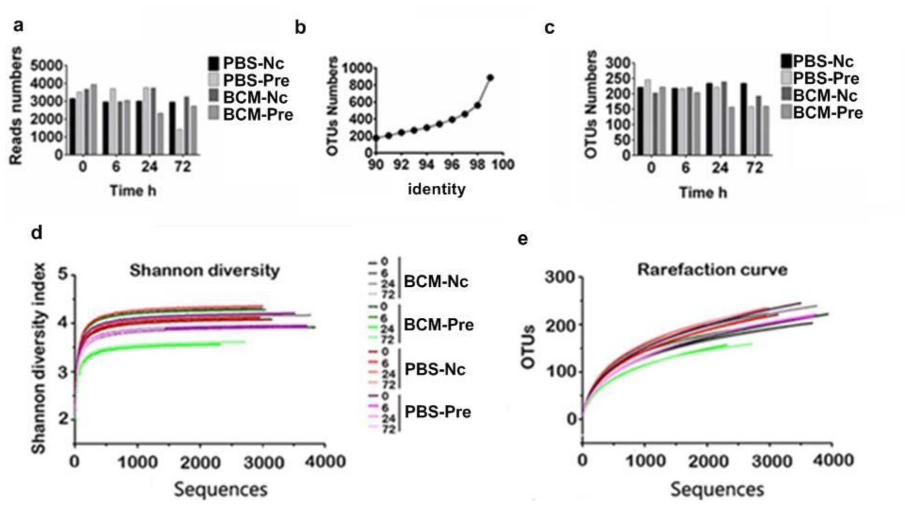

Supplementary Figure 1 **Operational taxonomic units (OTUs) delineation and distribution, and evaluation of the sequencing depth in each sample.** (a) Numbers of high quality bacteria reads in each sample. (b) The numbers of OTUs identified at several different similarity levels. The x-axis shows the identity, the y-axis shows the numbers of OTUs. (c) OTU numbers in each sample. (d) Shannon diversity index curves of all samples. (e) Rarefaction curves of all samples. "Pre" means Prebiotic cultures, and "Nc" means negative control cultures.

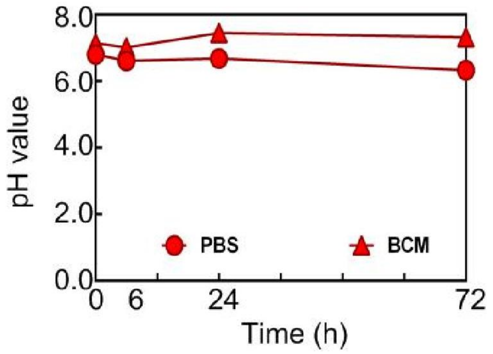

Supplementary Figure 2 Variations of pH in the negative control cultures of both PBS and BCM systems.

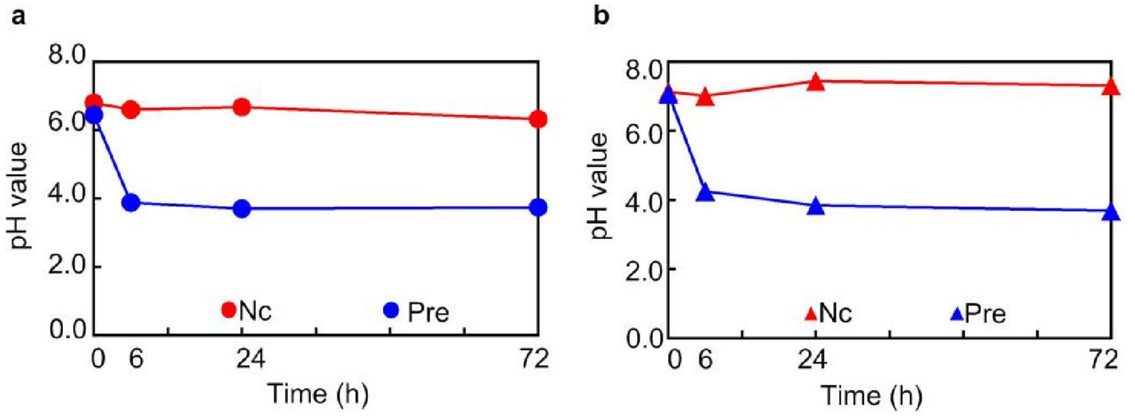

Supplementary Figure 3 Variations of pH during the prebiotic fermentation in the PBS (a) and BCM (b) systems. “Pre” means prebiotic cultures, and “Nc” means negative control cultures.

Supplementary Table S1 **The phylogeny and relative abundance of the 20 key OTUs altered in the PBS negative control (no prebiotic) fermentations over time.** Samples of "Pre0" and "Nc0", representing the baseline samples in prebiotic and negative control groups respectively, were constrained into the "Baseline" group in redundancy analysis, whereas "Nc6", "Nc24" and "Nc72" were constrained into the "after" fermentation group. In addition, the 0, 6, 24 and 72 hours time points were constrained into the nominal variables as well. The abundance is marked with the color of the colorbar from blue to white then to red. "Fitness in Axis 1" represents the percentage of the variability in their values explained by the first axis. Key OTUs are identified at the family and genus level, with different colors representing different phyla: blue, *Firmicutes*; brown, *Bacteroidetes*; green, *Actinobacteria;* and red, *Proteobacteria*.

OTU No.	Samples/Relative abundance (%)					Fitness	Identification	
	Pre0	Nc0	Nc6	Nc24	Nc72	in Axis 1	Family	Genus
OTU147	6.02	9	8.45	3.98	2.01	89.10%	*Ruminococcaceae*	*Faecalibacterium*
OTU380	1.8	2.68	2.48	1.71	0.44	95.60%	*Ruminococcaceae*	
OTU550	3.06	4.66	4.24	1.81	0.85	89.20%	*Ruminococcaceae*	*Faecalibacterium*
OTU231	4.57	6.99	7.23	4.02	1.77	90.40%	*Ruminococcaceae*	*Faecalibacterium*
OTU207	1.4	1.44	1.93	0.5	0.17	92.50%	*Ruminococcaceae*	*Clostridium* IV
OTU73	2.06	1.5	1.63	1.74	0	93.20%	*Lachnospiraceae*	*Lachnospiraceae incertae sedis*
OTU94	5.14	4.34	3.94	3.08	0.44	97.50%	*Prevotellaceae*	*Prevotella*
OTU436	1.71	1.63	1.32	0.77	0	97.00%	*Lachnospiraceae*	*Lachnospiraceae incertae sedis*
OTU401	1.48	1.5	1.46	1.41	2.24	86.70%	*Porphyromonadaceae*	*Parabacteroides*
OTU167	1.51	0.8	0.95	0.9	2.01	52.50%	*Ruminococcaceae*	*Faecalibacterium*
OTU258	1.31	0.8	0.71	0.87	2.28	72.10%	*Bacteroidaceae*	*Bacteroides*
OTU257	10.48	9.7	9.03	10.04	11.53	69.40%	*Bacteroidaceae*	*Bacteroides*
OTU508	1.34	1.12	1.32	1.77	2.07	75.20%	*Porphyromonadaceae*	*Odoribacter*
OTU415	0.57	0.48	0.65	1.1	1.36	72.30%	*Ruminococcaceae*	
OTU118	0.66	0.42	1.12	1.34	2.11	58.00%	*Ruminococcaceae*	*Oscillibacter*
OTU374	0.8	0.77	0.92	1.24	2.75	96.60%	*Rikenellaceae*	*Alistipes*
OTU130	1.2	1.15	1.32	1.54	1.53	50.10%	*Ruminococcaceae*	*Oscillibacter*
OTU510	2.86	3.32	3.12	3.61	4.59	89.00%	*Ruminococcaceae*	
OTU135	0.49	0.77	1.05	2.34	4.01	72.80%	*unclassified bacteria*	
OTU414	0.86	0.86	1.12	1.2	1.97	86.60%	*Porphyromonadaceae*	*Barnesiella*

Supplementary Table S2 **The phylogeny and relative abundance of the 35 key OTUs altered in the BCM negative control (no prebiotic) fermentations over time.** Samples of "Pre0" and "Nc0", representing the baseline samples in prebiotic and negative control groups respectively, were constrained into the "Baseline" group in redundancy analysis, whereas "Nc6", "Nc24" and "Nc72" were constrained into the "after" fermentation group. In addition, the 0, 6, 24 and 72 hours time points were constrained into the nominal variables as well. The abundance is marked with the color of the colorbar from blue to white then to red. "Fitness in Axis 1" represents the percentage of the variability

in their values explained by the first axis. Key OTUs are identified at the family and genus level, with different colors representing different phyla: blue, *Firmicutes*; brown, *Bacteroidetes*; green, *Actinobacteria;* and red, *Proteobacteria*.

OTU	Samples/Relative abundance (%)					Fitness	Identification	
No.	Pre0	Nc0	Nc6	Nc24	Nc72	in Axis 1	Family	Genus
OTU258	1.35	0.22	0.13	0.24	0.03	68.70%	*Bacteroidaceae*	*Bacteroides*
OTU147	1.25	0.92	0.44	1.2	0.03	69.20%	*Ruminococcaceae*	*Faecalibacterium*
OTU242	1.12	0.73	1.54	2.51	0	56.00%	*Rikenellaceae*	*Alistipes*
OTU107	0.31	0.65	1.14	1.17	0	53.00%	*Lachnospiraceae*	*Coprococcus*
OTU457	1.22	1.41	1.07	0.99	0.37	87.10%	*Bacteroidaceae*	*Bacteroides*
OTU451	1.63	2.47	1.37	1.28	0.65	88.90%	*Lachnospiraceae*	*Lachnospiraceae incertae sedis*
OTU58	6.1	6.54	3.48	0.75	0.28	93.70%	*Lachnospiraceae*	*Roseburia*
OTU341	3.69	4.1	0.91	0.08	0	96.20%	*Ruminococcaceae*	*Faecalibacterium*
OTU390	0.56	1.03	0.24	0.03	0	95.70%	*Lachnospiraceae*	*Coprococcus*
OTU267	2.87	3.64	0.74	0.05	0	96.40%	*Ruminococcaceae*	*Faecalibacterium*
OTU172	2.49	2.8	0.34	0.08	0	98.30%	*Ruminococcaceae*	*Faecalibacterium*
OTU207	1.12	1.17	0	0	0	69.20%	*Ruminococcaceae*	*Clostridium* IV
OTU17	7.12	7.9	3.12	0.03	0.06	71.10%	*Lachnospiraceae*	*Coprococcus*
OTU430	0.56	1.44	0.17	0	0	80.60%	*Lachnospiraceae*	*Lachnospiraceae incertae sedis*
OTU257	13.17	9.93	6.83	2.88	3.13	79.10%	*Bacteroidaceae*	*Bacteroides*
OTU351	7.76	4.97	0.84	0.03	0.03	82.50%	*Lachnospiraceae*	*Roseburia*
OTU550	2.44	2.2	0.5	0.37	0	89.30%	*Ruminococcaceae*	*Faecalibacterium*
OTU161	0.76	0.84	1.41	0.03	0.03	62.40%	*Bacteroidaceae*	*Bacteroides*
OTU425	2.31	5.21	3.59	1.31	0.03	79.10%	*Lachnospiraceae*	
OTU380	2.42	4.13	3.89	2.75	0	68.70%	*Ruminococcaceae*	
OTU198	0.2	0.35	1.47	1.71	2.06	76.30%	*Coriobacteriaceae*	*Asaccharobacter*
OTU471	0.41	0.73	2.35	5.84	6.48	83.40%	*Lachnospiraceae*	*Clostridium* XlVa
OTU150	0	0.11	0.91	1.47	2.83	59.90%	*Acidaminococcaceae*	*Phascolarctobacterium*
OTU365	0.2	0.33	2.45	2.88	2.89	71.30%	*Coriobacteriaceae*	*Asaccharobacter*
OTU51	1.42	3.37	3.08	7.33	11.39	79.60%	*Veillonellaceae*	*Dialister*
OTU250	0.03	0	0.13	3.76	6.88	80.10%	*Desulfovibrionaceae*	*Bilophila*
OTU496	0.08	0	0.13	0.99	3.47	71.50%	*Lachnospiraceae*	*Clostridium* XlVa
OTU162	0.1	0.05	0.2	0.67	3.19	96.60%	*Lachnospiraceae*	*Clostridium* XlVa
OTU240	0	0	0.17	0.51	2.58	89.90%	*Lachnospiraceae*	*Clostridium* XlVa
OTU481	0	0	0.1	0.56	2.06	90.40%	*Lachnospiraceae*	*Clostridium* XlVa
OTU424	0	0	0.03	0.08	1.47	98.40%	*Ruminococcaceae*	*Oscillibacter*
OTU204	0.15	0.24	0.4	3.55	1.26	56.60%	*Enterobacteriaceae*	*Escherichia/Shigella*
OTU130	0.33	0.41	4.32	11.59	4.85	62.00%	*Ruminococcaceae*	*Oscillibacter*
OTU325	0.03	0	0.5	1.15	1.11	65.00%	*Ruminococcaceae*	
OTU239	0.1	0.19	3.69	1.49	3.62	69.50%	*Streptococcaceae*	*Streptococcus*

Supplementary Table S3 The phylogeny and relative abundance of the 31 key OTUs increased or decreased in the PBS system after fermentation with addition of the prebiotic over time. Groups of "Pre" and "Nc", representing samples from the prebiotic and negative control cultures respectively, were constrained into paired groups as the non-nominal variables in redundancy analysis, and the 0, 6, 24 and 72 hours time points were constrained into the nominal variables. The abundance is marked with the color of the colorbar from blue to white then to red. "Fitness in Axis 1 and Axis 2" represent the percentages of the variability in their values explained by the first and second axis. Key OTUs are identified at the family and genus level, with different colors representing different phyla: blue, *Firmicutes*; brown, *Bacteroidetes*.

OTU	Relative abundance (%)								Fitness		Identification	
No.	Nc0	Nc6	Nc24	Nc72	Pre0	Pre6	Pre24	Pre72	in Axis 1	in Axis 2	Family	Genus
OTU215	0.54	0.31	0.10	0.03	0.80	3.71	3.04	1.82	13.9%	64.8%	*Lachnospiraceae*	*Roseburia*
OTU351	0.54	0.41	0.23	0.03	0.63	2.41	2.40	2.10	11.2%	57.9%	*Lachnospiraceae*	*Roseburia*
OTU58	0.29	0.31	0.47	0.24	0.60	1.08	2.21	0.42	11.6%	48.6%	*Lachnospiraceae*	*Roseburia*
OTU25	1.63	1.49	2.11	0.88	2.03	3.71	2.19	2.17	3.5%	59.3%	*Ruminococcaceae*	*Ruminococcus*
OTU550	4.66	4.24	1.81	0.85	3.06	5.26	4.90	4.41	0.0%	53.2%	*Ruminococcaceae*	*Faecalibacterium*
OTU147	9.00	8.45	3.98	2.01	6.02	9.26	10.39	8.82	0.0%	49.5%	*Ruminococcaceae*	*Faecalibacterium*
OTU425	1.79	1.09	0.50	0.61	1.03	1.52	3.30	1.26	2.8%	35.0%	*Lachnospiraceae*	
OTU231	6.99	7.23	4.02	1.77	4.57	7.58	7.94	5.39	3.0%	50.7%	*Ruminococcaceae*	*Faecalibacterium*
OTU94	4.34	3.94	3.08	0.44	5.14	1.82	6.26	5.81	0.0%	34.3%	*Prevotellaceae*	*Prevotella*
OTU380	2.68	2.48	1.71	0.44	1.80	1.44	3.33	2.52	1.2%	30.4%	*Ruminococcaceae*	
OTU108	0.10	0.03	0.13	0.24	0.09	0.76	6.45	1.89	59.8%	3.2%	*Lachnospiraceae*	*Blautia*
OTU172	0.29	0.48	0.54	0.44	0.46	1.44	0.83	1.75	59.4%	6.1%	*Ruminococcaceae*	*Faecalibacterium*
OTU267	0.86	0.92	0.50	1.12	1.03	1.87	1.39	3.15	67.4%	3.5%	*Ruminococcaceae*	*Faecalibacterium*
OTU341	2.36	2.85	3.01	5.88	4.00	6.93	5.81	11.28	92.6%	0.0%	*Ruminococcaceae*	*Faecalibacterium*
OTU167	0.80	0.95	0.90	2.01	1.51	2.30	1.92	4.76	93.9%	0.1%	*Ruminococcaceae*	*Faecalibacterium*
OTU180	0.00	0.00	0.00	0.00	0.00	0.00	0.00	1.47	49.9%	3.1%	*Lactobacillaceae*	*Lactobacillus*
OTU436	1.63	1.32	0.77	0.00	1.71	1.63	1.17	0.21	11.8%	63.4%	*Lachnospiraceae*	*Lachnospiraceae incertae sedis*
OTU73	1.50	1.63	1.74	0.00	2.06	1.95	1.73	0.98	4.4%	55.5%	*Lachnospiraceae*	*Lachnospiraceae incertae sedis*
OTU207	1.44	1.93	0.50	0.17	1.40	0.38	0.05	0.00	78.9%	4.4%	*Ruminococcaceae*	*Clostridium* IV
OTU430	0.19	0.27	1.10	0.27	0.34	1.06	0.61	0.00	36.5%	6.3%	*Lachnospiraceae*	*Lachnospiraceae incertae sedis*
OTU224	1.79	1.32	1.84	1.05	1.57	1.92	0.85	0.49	62.8%	8.0%	*Bacteroidaceae*	*Bacteroides*

OTU457	1.76	1.56	1.81	2.55	1.26	0.98	0.88	1.19	16.2%	68.0%	*Bacteroidaceae*	*Bacteroides*
OTU510	3.32	3.12	3.61	4.59	2.86	0.65	0.61	1.54	22.4%	39.3%	*Ruminococcaceae*	
OTU242	3.51	4.04	4.42	5.81	4.23	2.38	1.47	1.54	36.7%	17.7%	*Rikenellaceae*	*Alistipes*
OTU374	0.77	0.92	1.24	2.75	0.80	0.52	0.24	0.56	7.5%	54.0%	*Rikenellaceae*	*Alistipes*
OTU135	0.77	1.05	2.34	4.01	0.49	0.16	0.11	0.14	28.3%	49.2%	*unclassified bacteria*	
OTU415	0.48	0.65	1.10	1.36	0.57	0.05	0.00	0.00	54.0%	8.6%	*Ruminococcaceae*	
OTU51	1.66	2.07	1.54	2.28	1.31	0.22	0.21	0.21	49.5%	22.5%	*Veillonellaceae*	*Dialister*
OTU401	1.50	1.46	1.41	2.24	1.48	1.06	0.72	0.49	44.3%	10.4%	*Porphyromonadaceae*	*Parabacteroides*
OTU102	0.51	0.81	1.20	1.22	0.51	0.73	0.48	0.63	1.8%	52.1%	*Rikenellaceae*	*Alistipes*
OTU118	0.42	1.12	1.34	2.11	0.66	0.46	0.67	0.42	4.3%	43.2%	*Ruminococcaceae*	*Oscillibacter*

Supplementary Table S4 The phylogeny and relative abundance of the 39 key OTUs increased or decreased in the BCM system after fermentation with addition of the prebiotic over time. Groups of "Pre" and "Nc", representing samples from the prebiotic and negative control cultures respectively, were constrained into paired groups as the non-nominal variables in redundancy analysis, and the 0, 6, 24 and 72 hours time points were constrained into the nominal variables. The abundance is marked with the color of the colorbar from blue to white then to red. "Fitness in Axis 1 and Axis 2" represent the percentages of the variability in their values explained by the first and second axis. Key OTUs are identified at the family and genus level, with different colors representing different phyla: blue, *Firmicutes*; brown , *Bacteroidetes;* Green, *Actinobacteria* and red, *Proteobacteria*.

OTU No.	Samples/Relative abundance (%)								Fitness		Identification	
	Nc0	Nc6	Nc24	Nc72	Pre0	Pre6	Pre24	Pre72	in Axis 1	in Axis 2	Family	Genus
OTU198	0.35	1.47	1.71	2.06	0.20	0.69	0.17	0.11	9.5%	48.9%	*Coriobacteriaceae*	*Asaccharobacter*
OTU365	0.33	2.45	2.88	2.89	0.20	0.50	0.17	0.15	17.0%	48.2%	*Coriobacteriaceae*	*Asaccharobacter*
OTU471	0.73	2.35	5.84	6.48	0.41	1.58	0.35	0.37	21.0%	36.6%	*Lachnospiraceae*	*Clostridium* XlVa
OTU130	0.41	4.32	11.59	4.85	0.33	0.99	0.39	0.04	2.3%	50.8%	*Ruminococcaceae*	*Oscillibacter*
OTU240	0.00	0.17	0.51	2.58	0.00	0.00	0.00	0.00	39.9%	30.0%	*Lachnospiraceae*	*Clostridium* XlVa
OTU162	0.05	0.20	0.67	3.19	0.10	0.10	0.00	0.04	22.6%	22.6%	*Lachnospiraceae*	*Clostridium* XlVa
OTU544	0.43	1.61	0.48	0.95	0.15	0.20	0.04	0.18	16.1%	55.2%	*Ruminococcaceae*	*Oscillibacter*
OTU204	0.24	0.40	3.55	1.26	0.15	0.26	0.26	0.26	32.6%	20.6%	*Enterobacteriaceae*	*Escherichia/Shigella*
OTU150	0.11	0.91	1.47	2.83	0.00	0.79	0.30	0.59	39.6%	3.6%	*Acidaminococcaceae*	*Phascolarctobacterium*
OTU239	0.19	3.69	1.49	3.62	0.10	1.75	0.26	0.07	6.2%	27.9%	*Streptococcaceae*	*Streptococcus*

OTU271	0.57	2.65	1.25	0.46	0.46	0.26	0.78	0.18	1.6%	53.4%	*Ruminococcaceae*	
OTU250	0.00	0.13	3.76	6.88	0.03	0.20	0.09	0.22	40.1%	0.9%	*Desulfovibrionaceae*	*Bilophila*
OTU170	0.73	0.07	2.13	4.85	1.30	1.49	1.43	1.92	16.8%	20.4%	*Desulfovibrionaceae*	*Lawsonia*
OTU425	5.21	3.59	1.31	0.03	2.31	3.56	3.81	0.74	74.4%	0.7%	*Lachnospiraceae*	
OTU267	3.64	0.74	0.05	0.00	2.87	1.98	2.03	1.92	59.0%	7.6%	*Ruminococcaceae*	*Faecalibacterium*
OTU224	0.57	0.20	0.00	0.00	0.46	0.03	1.17	0.22	34.5%	8.2%	*Bacteroidaceae*	*Bacteroides*
OTU550	2.20	0.50	0.37	0.00	2.44	2.18	1.17	2.51	55.1%	6.5%	*Ruminococcaceae*	*Faecalibacterium*
OTU341	4.10	0.91	0.08	0.00	3.69	4.39	3.63	4.64	55.1%	11.5%	*Ruminococcaceae*	*Faecalibacterium*
OTU172	2.80	0.34	0.08	0.00	2.49	2.54	2.21	2.73	55.0%	12.6%	*Ruminococcaceae*	*Faecalibacterium*
OTU380	4.13	3.89	2.75	0.00	2.42	3.17	3.03	4.02	48.7%	0.7%	*Ruminococcaceae*	
OTU242	0.73	1.54	2.51	0.00	1.12	2.08	0.91	2.43	42.1%	1.8%	*Rikenellaceae*	*Alistipes*
OTU78	0.35	0.20	0.59	0.25	0.38	0.76	0.61	1.55	0.1%	54.5%	*Lachnospiraceae*	*Blautia*
OTU258	0.22	0.13	0.24	0.03	1.35	0.13	4.24	2.62	9.3%	42.4%	*Bacteroidaceae*	*Bacteroides*
OTU51	3.37	3.08	7.33	11.39	1.42	5.91	12.80	17.05	46.2%	18.5%	*Veillonellaceae*	*Dialister*
OTU213	0.05	0.07	0.03	0.06	0.05	1.09	2.12	3.06	0.1%	67.8%	*Bifidobacteriaceae*	*Bifidobacterium*
OTU451	2.47	1.37	1.28	0.65	1.63	3.00	1.25	0.77	72.8%	5.3%	*Lachnospiraceae*	*Lachnospiraceae incertae sedis*
OTU147	0.92	0.44	1.20	0.03	1.25	1.58	0.91	0.41	71.5%	0.5%	*Ruminococcaceae*	*Faecalibacterium*
OTU71	0.16	0.50	0.40	0.00	0.56	4.88	0.09	0.07	69.2%	0.0%	*Lachnospiraceae*	*Dorea*
OTU332	0.14	1.24	0.11	0.03	0.66	2.24	0.22	0.00	57.2%	13.7%	*Lachnospiraceae*	*Lachnospiraceae incertae sedis*
OTU516	0.33	0.54	0.64	0.58	0.71	1.02	0.17	0.11	10.1%	19.1%	*Lachnospiraceae*	*Clostridium* XlVa
OTU107	0.65	1.14	1.17	0.00	0.31	2.54	0.04	0.07	53.3%	6.8%	*Lachnospiraceae*	*Coprococcus*
OTU538	0.16	1.31	0.21	0.00	0.13	0.26	0.00	0.00	37.1%	38.6%	*Lachnospiraceae*	*Lachnospiraceae incertae sedis*
OTU427	0.19	1.04	1.07	0.09	0.00	0.20	0.09	0.07	7.9%	27.2%	*Lachnospiraceae*	*Clostridium* XlVa
OTU430	1.44	0.17	0.00	0.00	0.56	0.07	0.00	0.00	47.0%	15.1%	*Lachnospiraceae*	*Lachnospiraceae incertae sedis*
OTU17	7.90	3.12	0.03	0.06	7.12	0.83	0.00	0.00	26.9%	33.5%	*Lachnospiraceae*	*Coprococcus*
OTU519	0.62	1.21	0.21	0.31	1.35	0.40	0.39	0.04	38.5%	26.2%	*Erysipelotrichaceae*	*Clostridium* XVIII
OTU58	6.54	3.48	0.75	0.28	6.10	0.17	0.00	0.04	4.0%	43.7%	*Lachnospiraceae*	*Roseburia*
OTU390	1.03	0.24	0.03	0.00	0.56	0.00	0.00	0.00	21.2%	41.4%	*Lachnospiraceae*	*Coprococcus*
OTU351	4.97	0.84	0.03	0.03	7.76	0.00	0.00	0.00	8.6%	40.0%	*Lachnospiraceae*	*Roseburia*
